# Supplementary material for: Peripheral membrane protein endophilin B1 probes, perturbs and permeabilizes lipid bilayers
Source: Commun Biol. 2025 Feb 5;8:182. doi: 10.1038/s42003-025-07610-1 (PMC11799418; doi:10.1038/s42003-025-07610-1)
Supplement: Supplementary file 1 — supplementary information [file 42003_2025_7610_MOESM1_ESM.pdf]

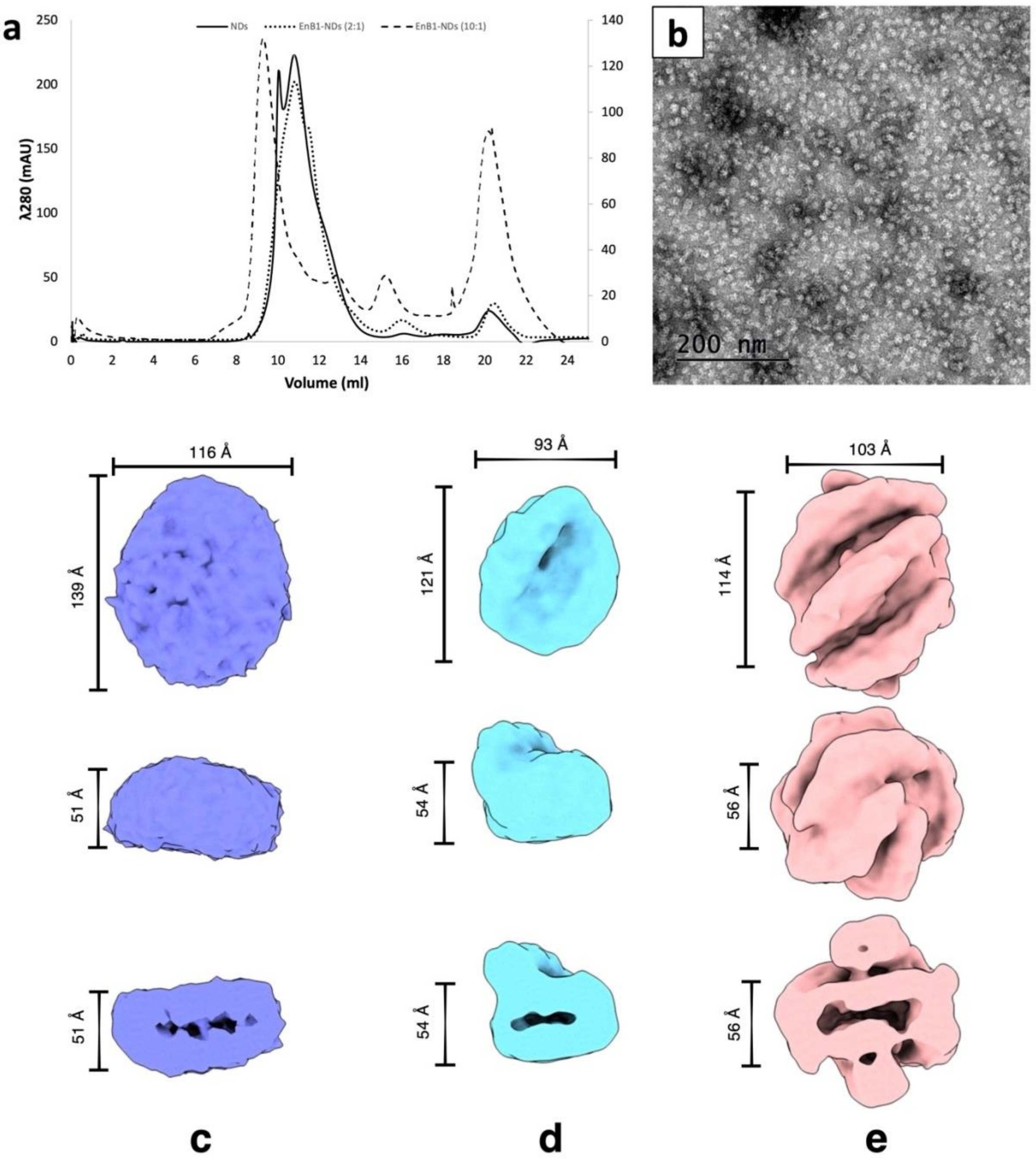

Supplementary Figure 1. Higher ratios of endophilin B1 (EnB1) yield nanodiscs (NDs) with more EnB1 decoration. **a**) SEC from Superdex 200 Increase 10/300 analysis of NDs, EnB1+NDs (1:2 of MSP2N2: EnB1), and EnB1+NDs (1:10 of MSP2N2: EnB1). **b**) Negative stain micrograph showing the EnB1 10:1 peak that eluted at 9.3 ml visualized by 1% uranyl acetate. Cryo-EM reconstructions of naked MSP2N2 NDs (**c**), EnB1 ND complex (1:2 of MSP2N2:EnB1) (**d**), and EnB1 ND complex (1:10 of MSP2N2:EnB1) (**e**) from Glacios data sets. EnB1 binding reduces the diameter of MSP2N2 NDs by roughly 20 Å and the binding of 6 EnB1 dimers changes the shape of the disc.

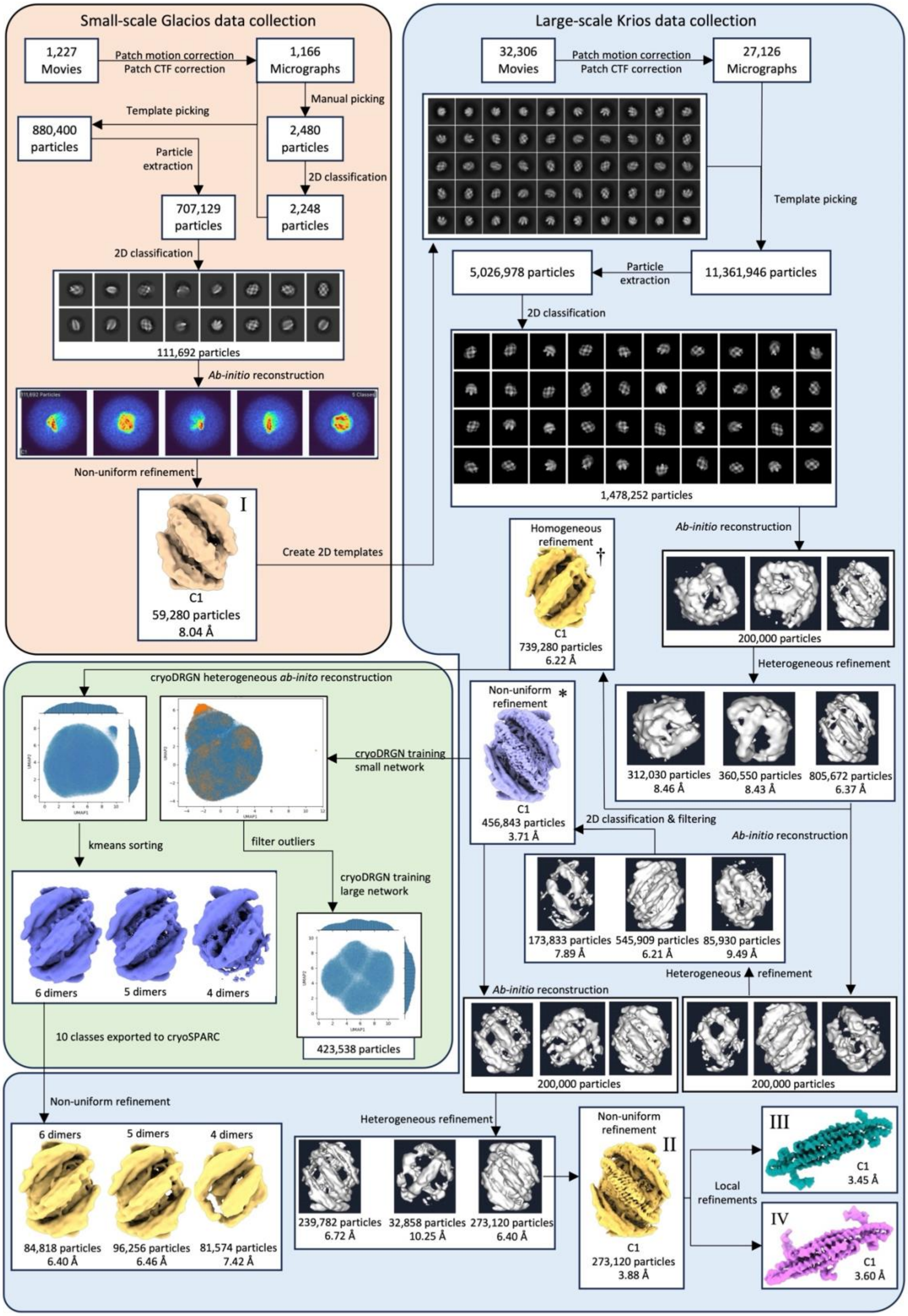

Supplementary figure 2. Cryo-EM workflow summary.

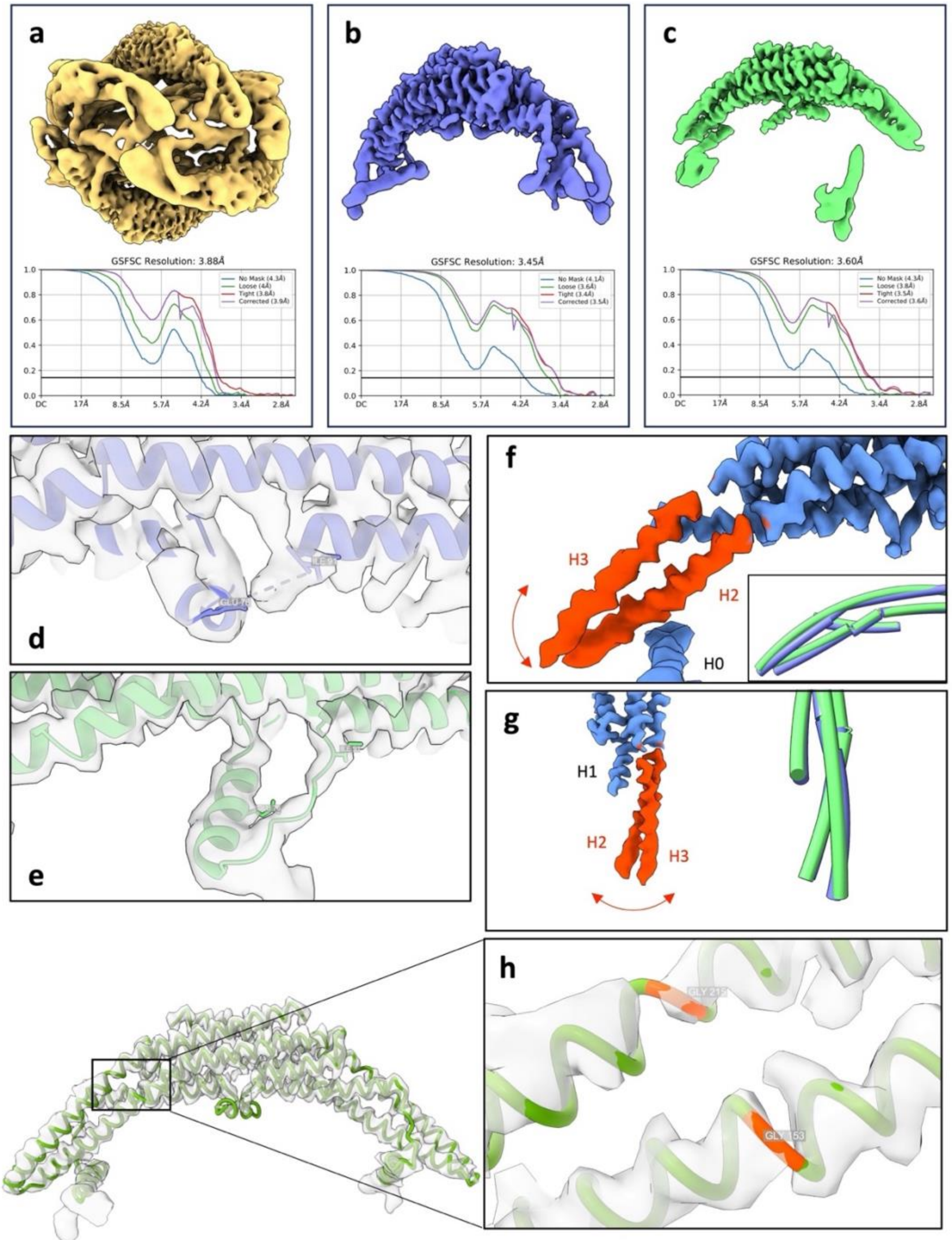

Supplementary figure 3. Cryo-EM maps and subsequent models reveal distinct conformations of amphipathic regions and the BAR domain. **a**) Final C1 consensus map and gold-standard Fourier-shell correlation (GSFSC) curve. **b**) Center dimer focused map and GSFSC curve. **c**) Side dimer focused map and GSFSC curve. **d**) Center dimer map and atomic model highlighting that H1i is disordered in solution. **e**) Side dimer map and atomic model highlighting that H1i assumes a mostly helical conformation when membrane-inserted **f-g**) The distal ends of H2 and H3 helices (red) have worse local resolution due to flexibility around the hinge region. The position of these is also different in the atomic models (center dimer in blue and side dimer in green). **h**) Center dimer map and model highlighting the two glycines, Gly153 and Gly215, that are responsible for the increased flexibility of the distal ends of the BAR domain.

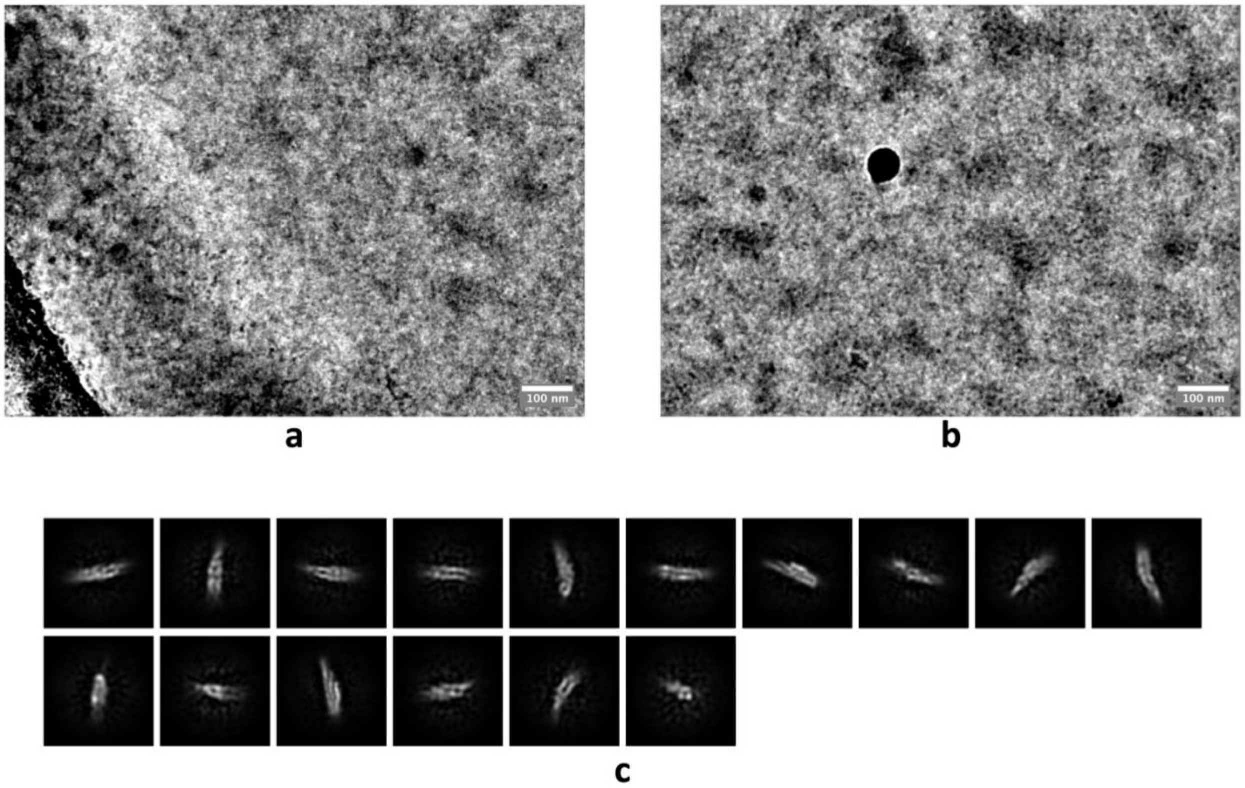

Supplementary figure 4. Cryo-EM of *wt* EnB1 in solution. **a-b**) Raw micrographs from high-resolution data collection of soluble EnB1, low-pass filtered to 8 Å. **c**) 2D classes of soluble EnB1 (43,341 particles).

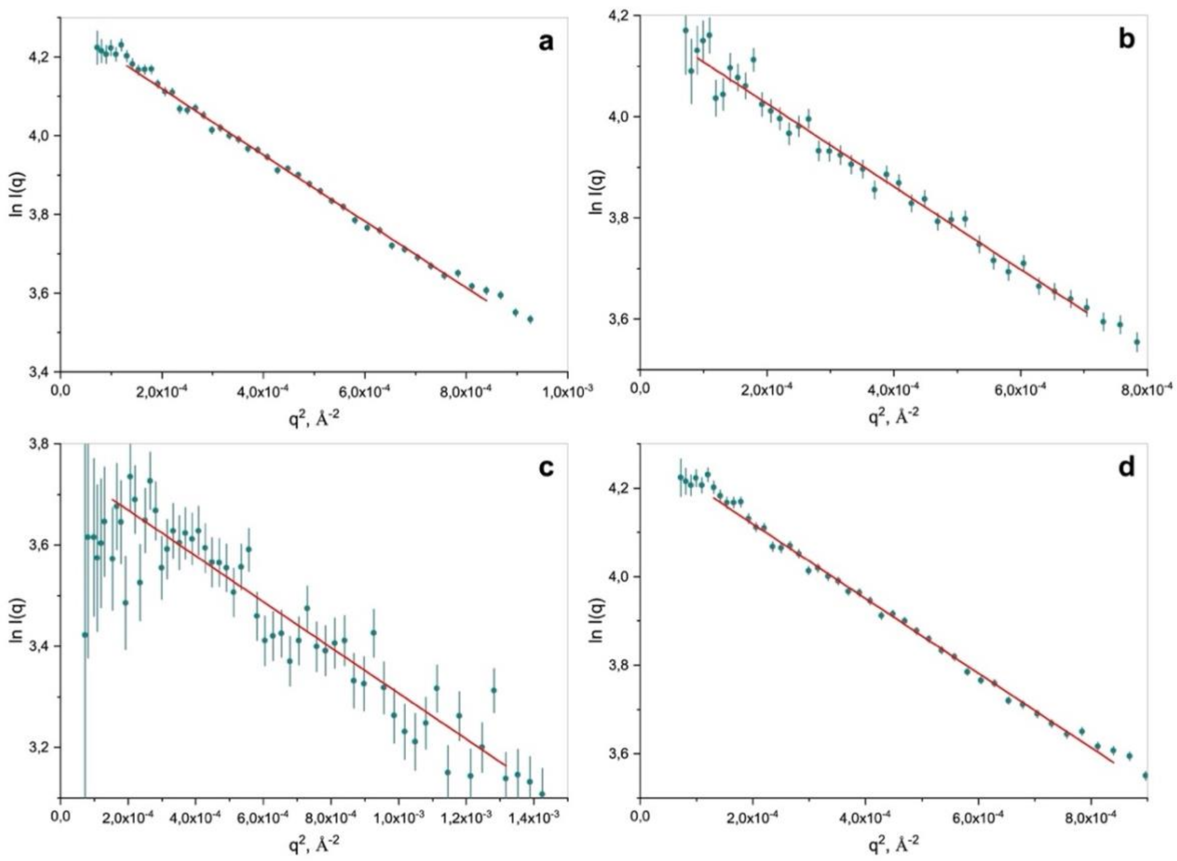

Supplementary Figure 5. Guinier approximations of SAXS data obtained for *wt* EnB1 at different concentrations: **a**) 0.69 mg/ml, **b**) 0.34 mg/ml and **c**) 0.14 mg/ml, and EnB1_ΔSH_3 at 2.5 mg/ml (**d**).

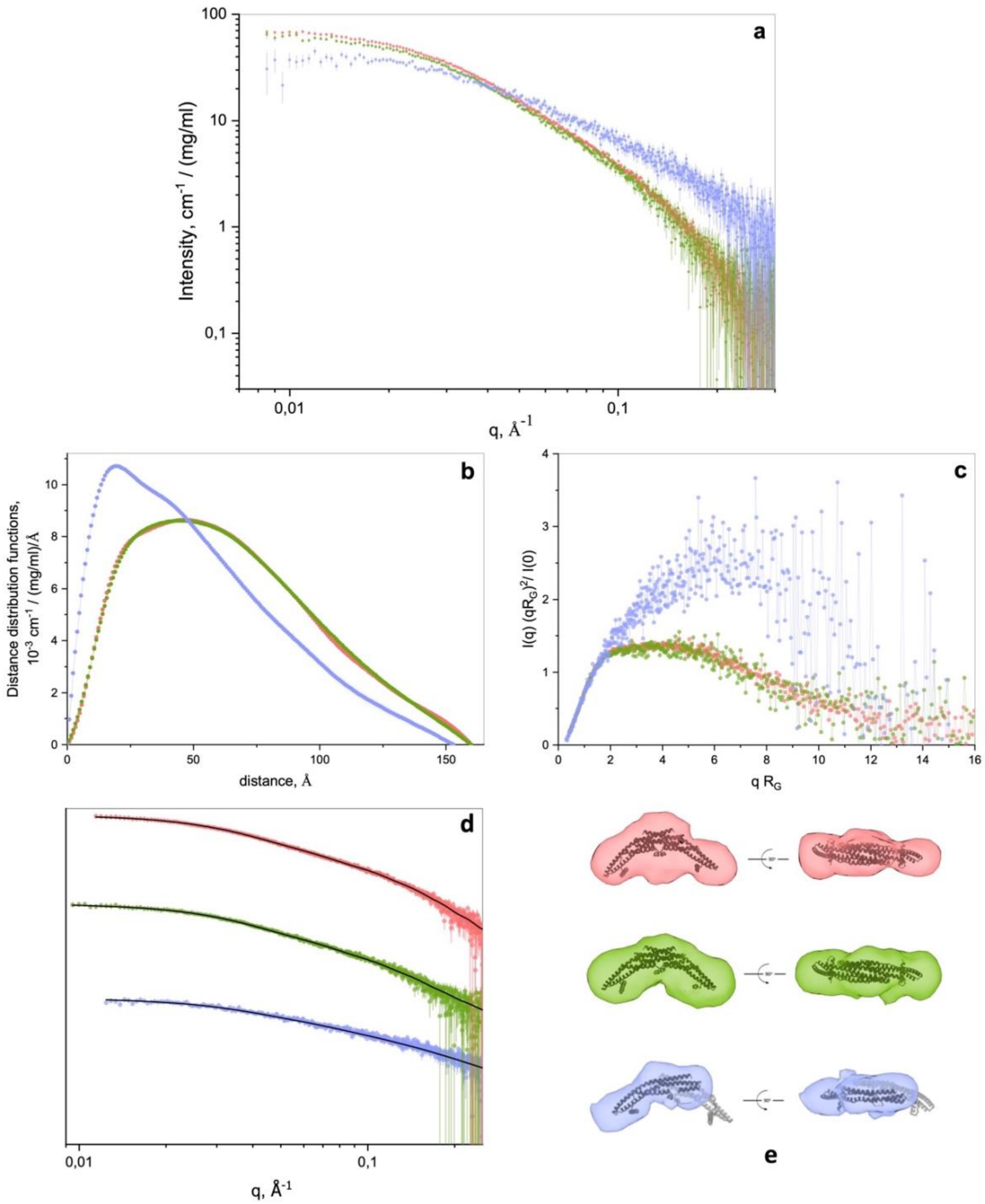

Supplementary Figure 6. SAXS curves for *wt* EnB1, corresponding distance distribution functions and Normalized Kratky plots. **a**) SAXS experimental data of *wt* EnB1 at concentrations 0.69 mg/ml (red), 0.34 mg/ml (green) and 0.14 mg/ml (blue). **b**) Distance distribution functions calculated from SAXS experimental data shown in (**a**). **c**) Normalized Kratky plots obtained from SAXS: I(0) and R_G values were calculated from *P(r).* **d**) Model fits (black lines) of data shown in (**a**) obtained from GNOM calculations and DENSSweb software. **e**) SAXS *ab-initio* reconstructions determined from SAXS data of *wt* EnB1 at different concentrations and comparisons of each with the cryo-EM center dimer atomic structure (black ribbons).

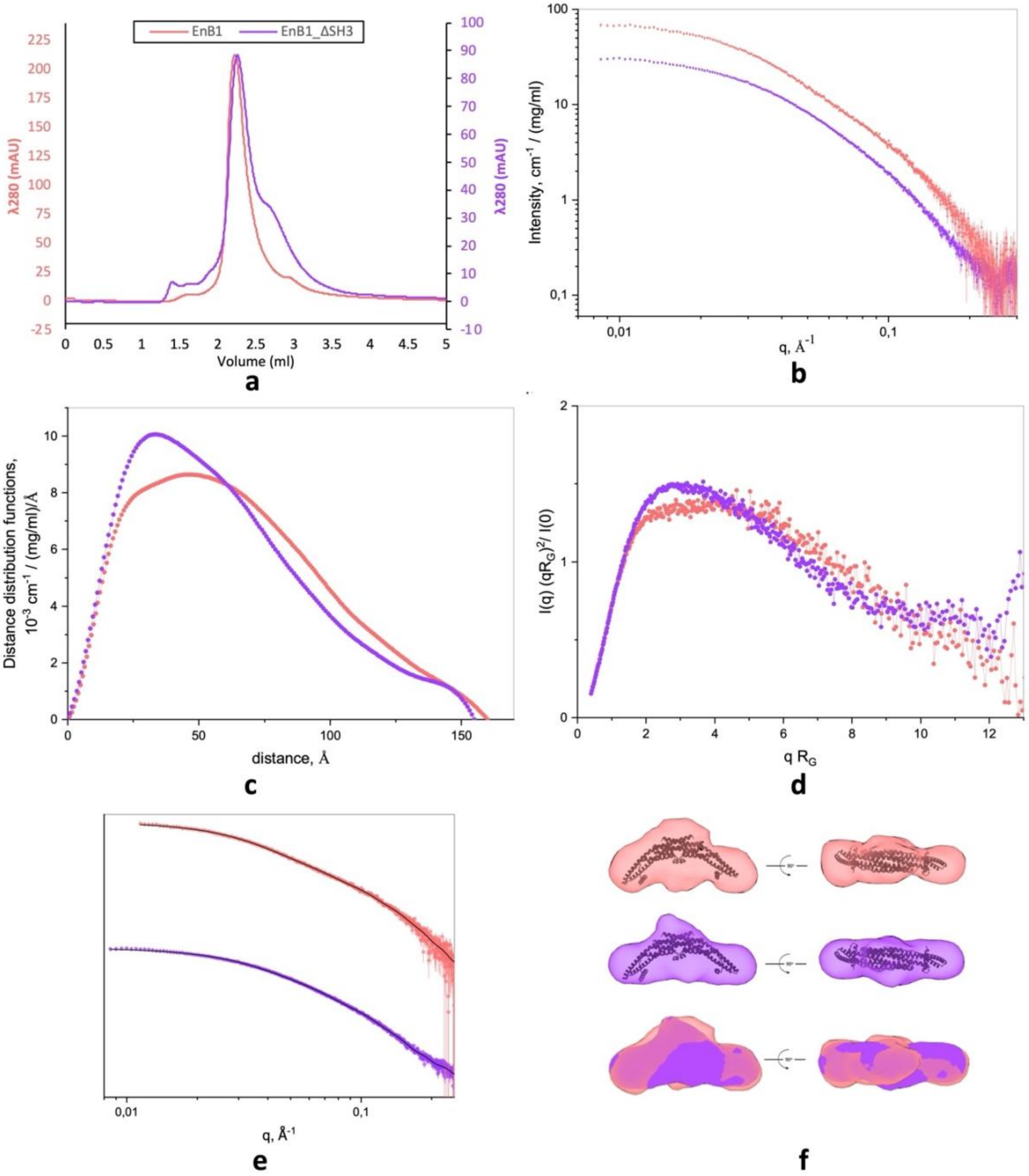

Supplementary figure 7. SEC chromatogram and SAXS curves from analysis of EnB1 and EnB1_ΔSH3. **a**) EnB1 (red) and EnB1_ΔSH3 (purple) elute at 2.24 ml and 2.27 ml, respectively, from an AdvanceBio SEC 300Å, 4.6 x 300 mm, 2.7 µm, HPLC column (Agilent). **b**) SAXS experimental data of EnB1 (red) and EnB1_ΔSH3 (purple). **c**) Distance distribution functions calculated from SAXS experimental data shown in (a). **d**) Normalized Kratky plots obtained from SAXS: I(0) and R_G values were calculated from *P(r)*. **e**) Model fits (black lines) for EnB1 and EnB1_ΔSH3 obtained from GNOM calculations and DENSSweb software. Electron density maps obtained for EnB1 and EnB1_ΔSH3, comparison of them with atomic structure (black ribbons). **f**) Comparison of SAXS *ab-initio* reconstructions.

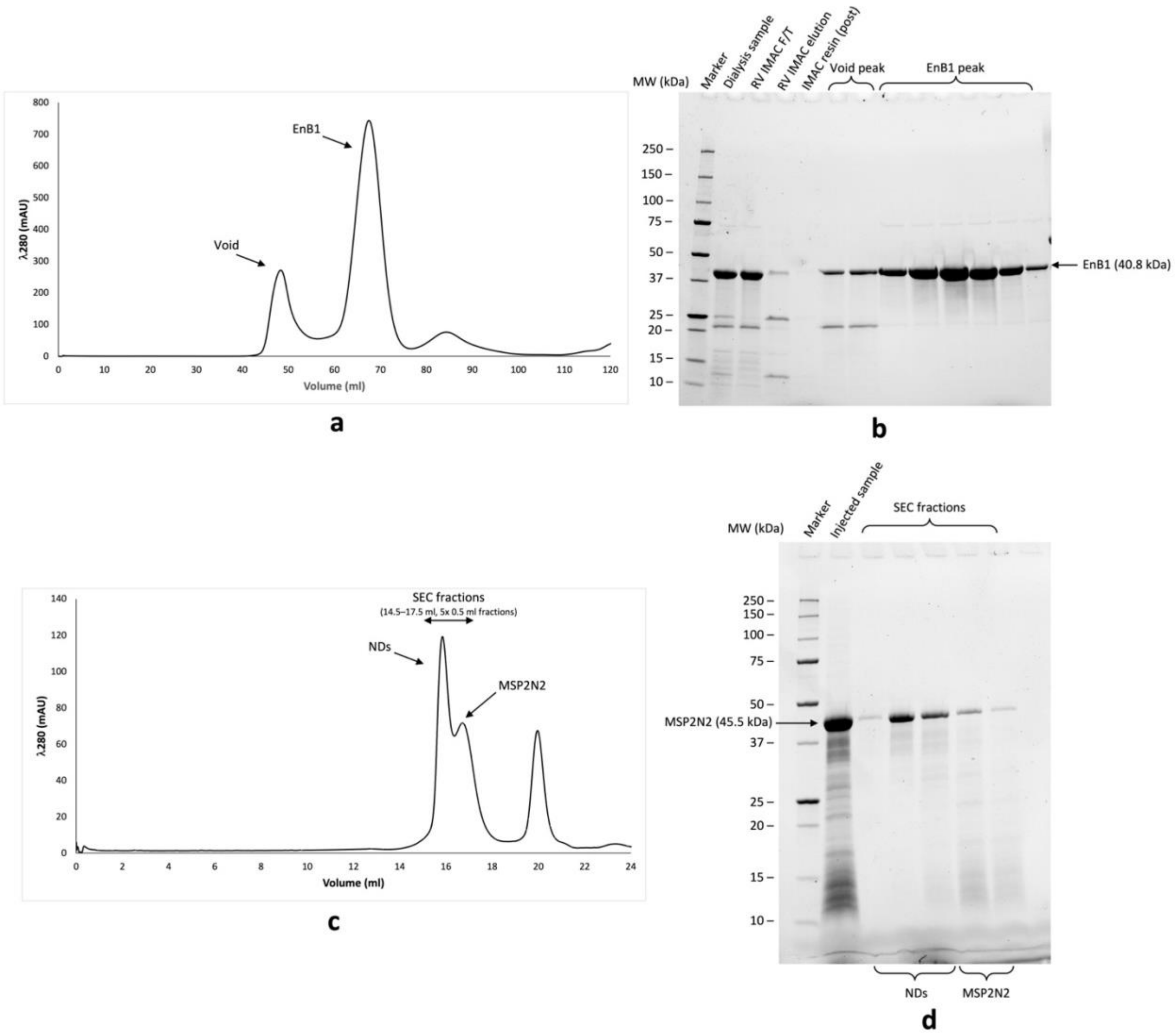

Supplementary figure 8. Purification of EnB1 and MSP2N2 NDs. **a**) Representative SEC chromatogram from analysis of EnB1 using a Superdex 200 Increase 16/600 column (GE Healthcare). **b**) Stain-free visualization (Bio-Rad) of SDS-PAGE analysis of SEC run shown in (a). **c**) Representative SEC chromatogram from analysis of MSP2N2 NDs using a Superose 6 10/300 GL (Cytiva) column. **d**) Stain-free visualization of SDS-PAGE analysis of SEC run shown in (**c**).

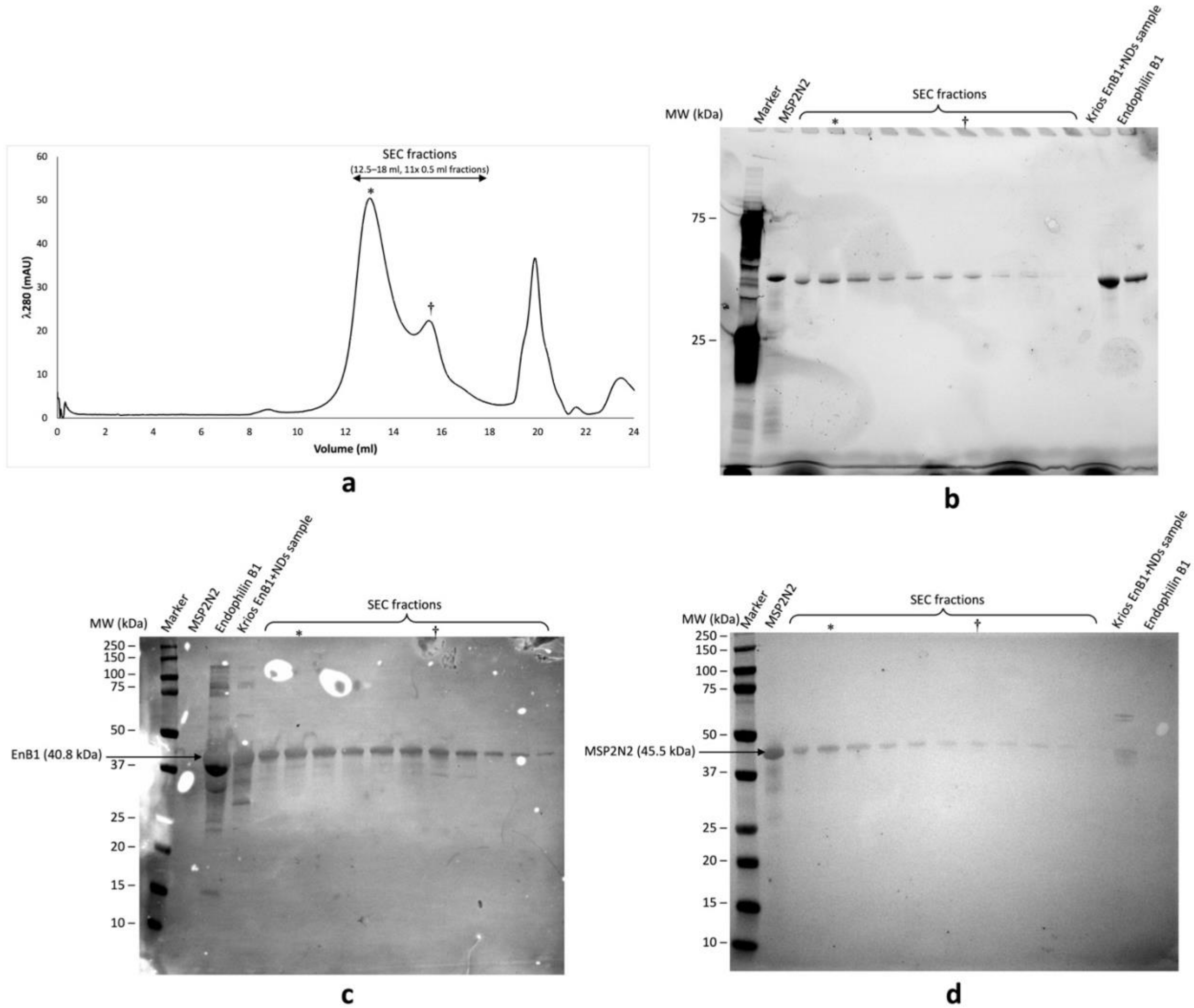

Supplementary figure 9. Results from a purification of EnB1 decorated NDs (1:10 MSP2N2: EnB1). **a**) Representative SEC chromatogram from analysis of EnB1 incubated with MSP2N2 NDs using a Superose 6 10/300 GL column. **b**) Stain-free visualization of SDS-PAGE analysis of peak fractions from SEC run shown in (a), purified EnB1 and MSP2N2 and a previous EnB1-decorated NDs sample used for cryo-EM data collection (Krios EnB1+NDs sample). Western blot analyses samples shown in (**b**) using anti-EnB1 (**c**) and anti-polyHis (**d**) primary antibodies and alkaline phosphatase conjugated secondary antibodies. Fractions from the dominant peak are marked with an asterisk (*) contain both EnB1 and MSP2N2. The sample used for the high-resolution Krios data collection also contains both EnB1 and MSP2N2.

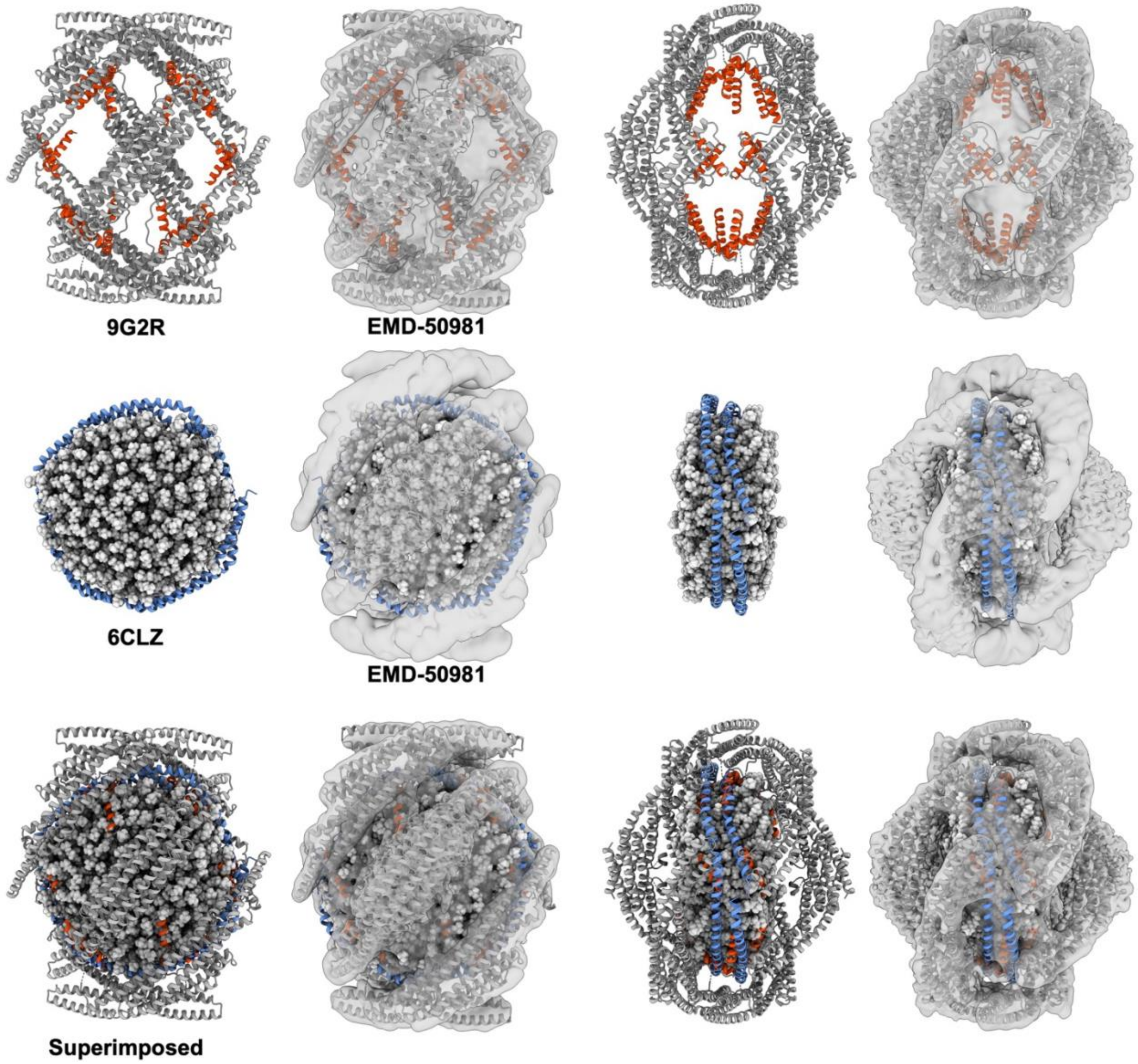

Supplementary figure 10. Top row: The structure of endophilin B1 decorated lipoprotein particles (PDB ID: 9G2R) docked into our consensus map of endophilin B1 decorated lipoprotein particles (EMD-50981) with the amphipathic motifs highlighted in red. Middle row: The NMR structure of MSP1D1 (shown in blue) nanodiscs (PDB ID: 6CLZ) docked into the consensus map of endophilin B1 decorated lipoprotein particles (EMD-50981). The bilayer in the 6CLZ structure has a similar diameter and width of the lipid bilayer as that displayed in our consensus map. Bottom row: The superimposed structures of endophilin B1 decorated lipoprotein particles (PDB ID: 9G2R) and NMR structure of MSP1D1 (PDB ID: 6CLZ) docked into the consensus map of endophilin b1 decorated lipoprotein particles (EMD-50981). Superimposing the two structures shows that the amphipathic motifs of endophilin B1 (red) in the lipoprotein complex occupy the same positions as MSP1D1 (blue) does in the nanodisc structure.

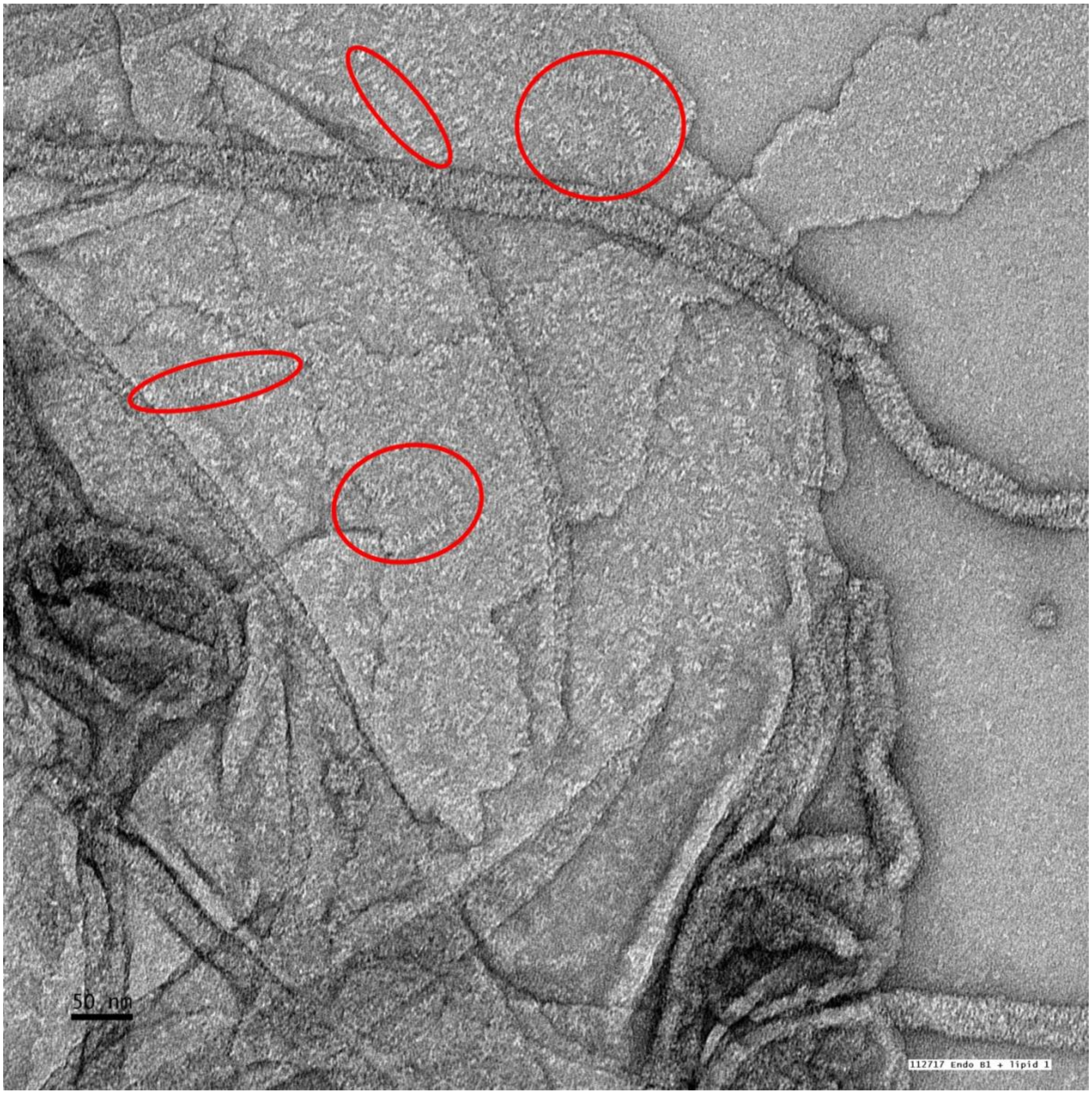

Supplementary Figure 11. TEM micrograph of cardiolipin-containing liposomes incubated with endophilin B1 visualized by 1% uranyl acetate. Examples of areas where endophilin B1 has assembled side-to-side on a flat patch of membrane is highlighted in red.
