# Supplementary material for: Peripheral membrane protein endophilin B1 probes, perturbs and permeabilizes lipid bilayers
Source: Commun Biol. 2025 Feb 5;8:182. doi: 10.1038/s42003-025-07610-1 (PMC11799418; doi:10.1038/s42003-025-07610-1)
Supplement: Supplementary file 2 — Description of Additional Supplementary Files [file 42003_2025_7610_MOESM2_ESM.pdf]

Description of additional supplementary data

File name: Supplementary Movie 1.

Description: H0 dynamics appear coupled to changes in the conformations of the BAR domain. The movie can be split into two parts. Both traverse the same UMAP vizualization of cryoDRGN results for endophilin B1 bound to lipid bicelles, but show the results from different angles. The first is angled towards the flatter side of the bicelle and highlights the movement of the BAR domains. The second is angled towards the highly curved edge of the bicelle and highlights the movements of the amphipathic motifs, H0 and H1i.

File name: Supplementary Movie 2.

Description: Traversal of the second principal component of variability (PC2) shown in Fig. 3a.

File name: Supplementary Movie 3.

Description: Graph traversal of the UMAP distribution shown in Fig. 4a.
