# Supplementary material for: Peripheral membrane protein endophilin B1 probes, perturbs and permeabilizes lipid bilayers
Source: Commun Biol. 2025 Feb 5;8:182. doi: 10.1038/s42003-025-07610-1 (PMC11799418; doi:10.1038/s42003-025-07610-1)
Supplement: Supplementary file 6 — Reporting Summary [file 42003_2025_7610_MOESM6_ESM.pdf]

nature portfolio

Corresponding author(s): Anna Sundborger-Lunna

Last updated by author(s): 01/20/2025

Reporting Summary

Nature Portfolio wishes to improve the reproducibility of the work that we publish. This form provides structure for consistency and transparency in reporting. For further information on Nature Portfolio policies, see our Editorial Policies and the Editorial Policy Checklist.

Statistics

For all statistical analyses, confirm that the following items are present in the figure legend, table legend, main text, or Methods section.

n/a	Confirmed	
☐	☒	The exact sample size (n) for each experimental group/condition, given as a discrete number and unit of measurement
☐	☒	A statement on whether measurements were taken from distinct samples or whether the same sample was measured repeatedly
☐	☒	The statistical test(s) used AND whether they are one- or two-sided<br>*Only common tests should be described solely by name; describe more complex techniques in the Methods section.*
☒	☐	A description of all covariates tested
☒	☐	A description of any assumptions or corrections, such as tests of normality and adjustment for multiple comparisons
☐	☒	A full description of the statistical parameters including central tendency (e.g. means) or other basic estimates (e.g. regression coefficient) AND variation (e.g. standard deviation) or associated estimates of uncertainty (e.g. confidence intervals)
☐	☒	For null hypothesis testing, the test statistic (e.g. F, t, r) with confidence intervals, effect sizes, degrees of freedom and P value noted<br>*Give P values as exact values whenever suitable.*
☒	☐	For Bayesian analysis, information on the choice of priors and Markov chain Monte Carlo settings
☒	☐	For hierarchical and complex designs, identification of the appropriate level for tests and full reporting of outcomes
☐	☒	Estimates of effect sizes (e.g. Cohen's d, Pearson's r), indicating how they were calculated

Our web collection on statistics for biologists contains articles on many of the points above.

Software and code

Policy information about availability of computer code

Data collection	EDNA pipeline for SAXS data collection, EPU for cryo-EM data collection
Data analysis	CryoSparc, CryoDRGN, ATSAS, BioXTAS RAW

For manuscripts utilizing custom algorithms or software that are central to the research but not yet described in published literature, software must be made available to editors and reviewers. We strongly encourage code deposition in a community repository (e.g. GitHub). See the Nature Portfolio guidelines for submitting code & software for further information.

Data

Policy information about availability of data

All manuscripts must include a data availability statement. This statement should provide the following information, where applicable:

- Accession codes, unique identifiers, or web links for publicly available datasets
- A description of any restrictions on data availability
- For clinical datasets or third party data, please ensure that the statement adheres to our policy

Raw data for figure 5 has been deposited to Figshare and can be downloaded via: 10.6084/m9.figshare.28239134. Motion corrected micrographs are available for download from EMPIAR with the public accession code: EMPIAR-12470. Additional data supporting the findings of this manuscript are available upon request.

Human research participants

Policy information about studies involving human research participants and Sex and Gender in Research.

Reporting on sex and gender	N/A
Population characteristics	N/A
Recruitment	N/A
Ethics oversight	N/A

Note that full information on the approval of the study protocol must also be provided in the manuscript.

Field-specific reporting

Please select the one below that is the best fit for your research. If you are not sure, read the appropriate sections before making your selection.

☒ Life sciences ☐ Behavioural & social sciences ☐ Ecological, evolutionary & environmental sciences

For a reference copy of the document with all sections, see nature.com/documents/nr-reporting-summary-flat.pdf

Life sciences study design

All studies must disclose on these points even when the disclosure is negative.

Sample size	We collected more than 1 million versions of our samples (unique particles) for our study.
Data exclusions	We did not exclude any data in this study.
Replication	We prepare our sample two separate times and collected data on both. These two data sets produced identical results.
Randomization	We did not randomize our data.
Blinding	We did not conduct our experiments blind.

Reporting for specific materials, systems and methods

We require information from authors about some types of materials, experimental systems and methods used in many studies. Here, indicate whether each material, system or method listed is relevant to your study. If you are not sure if a list item applies to your research, read the appropriate section before selecting a response.

Materials & experimental systems

n/a	Involved in the study	
☐	☒	Antibodies
☒	☐	Eukaryotic cell lines
☒	☐	Palaeontology and archaeology
☐	☒	Animals and other organisms
☒	☐	Clinical data
☒	☐	Dual use research of concern

Methods

n/a	Involved in the study	
☒	☐	ChIP-seq
☒	☐	Flow cytometry
☒	☐	MRI-based neuroimaging

Antibodies

Antibodies used	anti-Bif-1: PA1-9017 Thermo Fisher Scientific, anti-His: SAB130682 Sigma Aldrich, Goat IgG anti-Mouse: 605-305-002 Rockland Immunochemicals, Rabbit IgG anti-goat: 605-4502 Rockland Immunochemicals
Validation	Validated by vendor and confirmed with control proteins.

Animals and other research organisms

Policy information about studies involving animals; ARRIVE guidelines recommended for reporting animal research, and Sex and Gender in Research

Laboratory animals	N/A
Wild animals	N/A
Reporting on sex	N/A
Field-collected samples	N/A
Ethics oversight	N/A

Note that full information on the approval of the study protocol must also be provided in the manuscript.
